# Supplementary material for: Citri grandis Exocarpium Extract Alleviates Atherosclerosis in ApoE−/− Mice by Modulating the Expression of TGF-β1, PI3K, AKT1, PPAR-γ, LXR-α, and ABCA1
Source: Foods. 2025 Dec 11;14(24):4267. doi: 10.3390/foods14244267 (PMC12732995; doi:10.3390/foods14244267)
Supplement: Supplementary file 1 [file foods-14-04267-s001.zip › foods-3874678-supplementary.pdf]

Supporting Information

Citri grandis Exocarpium Extract Alleviates Atherosclerosis in ApoE-/- Mice by Modulating the Expression of TGF-β1, PI3K, AKT1, PPAR-γ, LXR-α, and ABCA1

Jing Xu †, Wen-Zhao Wen †, Jun-Hui Zhao, Jun-Rong Guo, Zhuo-Ya Zhang and Ping Xiong *

Pharmaceutical Engineering Specialty, South China Agricultural University, Guangzhou 510640, China; 17898488256@163.com (J.X.); 20223138216@stu.scau.edu.cn (W.-Z.W.); 18122454201@163.com (J.-H.Z.); 13556181173@163.com (J.-R.G.); zzy20233185085@stu.scau.edu.cn (Z.-Y.Z.).

*Correspondence: xp0000542003@scau.edu.cn (XP); Tel.: +0086-13533385789

Contents

Table S1 The cytostatic effect of CGE on HUVECs cells($\overline{x}$ ± SE，n=6)

CGE (mg/mL)	Cytostatic rate(%)
1.25	-5.80 ± 6.45
2.5	-1.24 ± 4.89
5	21.48 ± 8.40
10	81.69 ± 8.16
20	92.43 ± 0.67

Table S2 The cytostatic effect of cholesterol on HUVECs cells ($\overline{x}$ ± SE，n=6)

Cholesterol (μmol/mL)	Cytostatic rate(%)
0	0 ± 0
2.5	10.55 ± 3.86
5	23.56 ± 2.48
10	36.59 ± 2.21
20	47.82 ± 1.81

Table S3 The protective effects of CGE on cholesterol induced cell injury ($\overline{x}$ ± SE，n=6)

Groups	Cytostatic rate(%)
NC	100 ± 0

MOD	59.60 ± 1.44
CGE-L	74.47 ± 5.38*
CGE-M	78.98 ± 5.28*
CGE-H	81.35 ± 3.83**

Table S4 The effect of CGE on the cell apoptotic rate ($\bar{x}$ ± SE，n=6)

Groups	Cell apoptotic rate(%)
NC	0 ± 0**
MOD	43.146 ± 1.603
CGE-L	34.856 ± 3.511
CGE-M	29.140 ± 3.782**
CGE-H	21.820 ± 1.191**

Table S5 Cellular oil red O staining ($\bar{x}$ ± SE，n=6)

Groups	Cellular oil red O staining
NC	0.06 ± 0.01***
MOD	0.48 ± 0.01
CGE-L	0.34 ± 0.02**
CGE-M	0.16 ± 0.01***
CGE-H	0.12 ± 0.01***

Table S6 Quantitative analysis of the migrated distance ($\bar{x}$ ± SE，n=6)

Groups	Migrated distance (%)
NC	8.21 ± 0.43***
MOD	49.16 ± 1.37
CGE-L	40.93 ± 1.19**
CGE-M	23.44 ± 1.10***
CGE-H	14.74 ± 0.73***

Table S7 Quantitative analysis of the migration cells ($\overline{x}$ ± SE，n=6)

Groups	Migrated cells
NC	78.81 ± 3.43***
MOD	203.20 ± 4.83
CGE-L	152.72 ± 3.00***
CGE-M	131.57 ± 4.50***
CGE-H	94.27 ± 2.59***

Table S8 The effect of CGE on serum lipid four($\overline{x}$ ± SD，n=6)

Groups	Lipid four (mmol/L)			
	TC	TG	LDL-C	HDL-C
NC	4.67 ± 0.54**	1.57 ± 0.18**	1.32 ± 0.26**	1.98 ± 0.21
MOD	15.53 ± 0.05	5.52 ± 0.74	5.20 ± 0.62	1.85 ± 0.31
SV	9.46 ± 1.17**	3.41 ± 0.53**	2.51 ± 0.27**	2.04 ± 0.30
GCE-L	13.59 ± 0.54*	5.03 ± 0.41	4.39 ± 0.48*	2.23 ± 0.24
GCE-M	11.4 ± 1.47**	4.30 ± 0.33**	3.88 ± 0.47**	2.18 ± 0.23
GCE-H	9.96 ± 0.68**	3.54 ± 0.43**	3.50 ± 0.43**	2.17 ± 0.38

Table S9 The effect of CGE on serum ALT and AST($\overline{x}$ ± SE，n=6)

Groups	ALT/(U/L)	AST/(U/L)
NC	40.74±3.30**	51.03 ± 2.08**
MOD	108.04±2.30	129.98 ± 3.40
SV	52.80±1.95**	54.50 ± 1.86**
GCE-L	95.49±1.52**	103.43 ± 1.55**
GCE-M	84.19±2.15**	93.58 ± 2.44**
GCE-H	75.10±2.48**	88.90 ± 3.57**

Table S10 Lipid deposition area ratio of whole aorta($\overline{x}$ ± SE，n=6)

Groups	ORO area ratio (%)

NC	0.00 ± 0.00***
MOD	1.08 ± 0.05
SV	0.49 ± 0.04**
CGE-L	0.84 ± 0.06*
CGE-M	0.73 ± 0.06**
CGE-H	0.62 ± 0.04**

Table S11 Lipid deposition area ratio of the cross-section of the aortic roots($\bar{x}$ ± SE，n=6)

Groups	ORO area ratio (%)
NC	0.64 ± 0.07***
MOD	7.89 ± 0.30
SV	5.27 ± 0.15*
CGE-L	6.96 ± 0.18
CGE-M	5.60 ± 0.21*
CGE-H	3.53 ± 0.08**

Table S12 The area ratio of plaque collagen fiber of the aortic roots($\bar{x}$±SE，n=6)

Groups	Fiber area ratio (%)
NC	6.30 ± 0.23***
MOD	16.36 ± 0.38
SV	5.68 ± 0.24***
CGE-L	10.15 ± 0.23*
CGE-M	8.73 ± 0.28*
CGE-H	7.30 ± 0.13**

Table S13 liver oil red staining($\bar{x}$±SE，n=6)

Groups	ORO area ratio of mouse liver (%)
NC	4.01 ± 0.32
MOD	24.01 ± 0.62

SV	8.64 ± 0.29
CGE-L	14.79 ± 0.27
CGE-M	16.99 ± 0.57
CGE-H	3.29 ± 0.37

Table S14 Masson staining of liver(x̅±SE，n=6)

Groups	Fiber area ratio of mouse liver (%)
NC	0.58±0.06
MOD	8.96±0.19
SV	1.30±0.12
CGE-L	3.26±0.1
CGE-M	1.53±0.14
CGE-H	1.39±0.08

Table S15 BCA protein assay

Number	OD (λ=570nm)						
	BCA standard solution	NC	MOD	SV	CGE-L	CGE-M	CGE-H
1	0.951	0.814	0.915	0.920	0.733	0.781	0.903
2	0.657	0.831	0.720	0.727	0.769	0.914	0.956
3	0.487	0.690	0.905	0.911	0.803	0.830	0.894
4	0.420	0.795	0.852	0.866	0.893	0.915	0.799
5	0.383	0.848	0.760	0.767	1.099	0.971	0.917
6	0.362	1.014	0.920	0.939	0.938	0.856	0.849
7	0.349	0.864	0.915	0.904	0.868	0.830	0.734
8	——	0.871	0.910	0.927	1.029	0.928	0.810

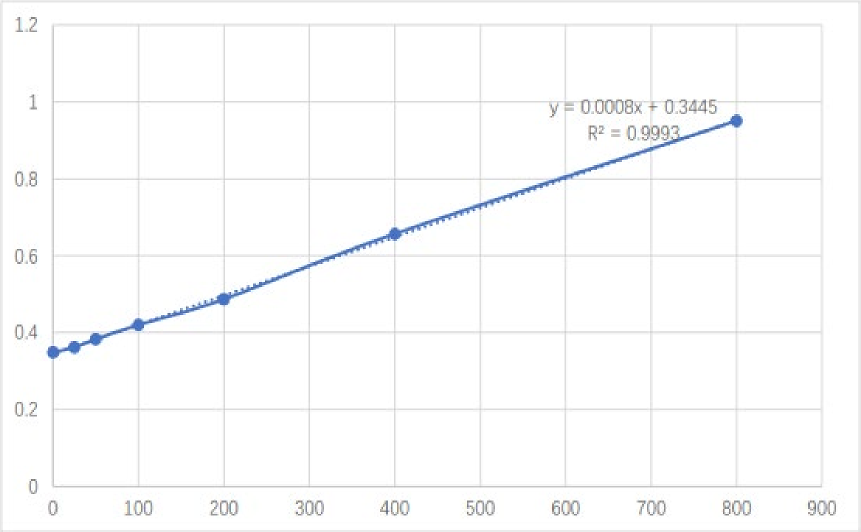

Figure S1 The standard curve established

The standard curve of BCA standard solution was drawn by protein concentration as the horizontal coordinate-axis X, and the absorbance values of samples as the vertical coordinate-axis Y. The regression equation was fitted as y=0.0008x+0.3445, the correlation of calibration curve was all good, which correlation coefficient (R^2) was equal to 0.9993.

Table S16 The loading quantity of protein sample

Number		Loading amount (μL)				
	NC	MOD	SV	GCE-L	GCE-M	GCE-H
1	6.4	5.3	5.2	7.7	6.9	5.4
2	6.2	7.8	7.8	7.1	5.3	4.9
3	8.7	5.4	5.3	6.5	6.2	5.5
4	6.7	5.9	5.8	5.5	5.3	6.6
5	6.0	7.2	7.1	4.0	4.8	5.2
6	4.5	5.2	5.0	5.1	5.9	5.9
7	5.8	5.5	5.4	5.7	6.2	7.7
8	5.7	5.3	5.2	4.4	5.1	6.4

The protein loading amount (μL) of each group was calculated by 30μg sample loading per well for electrophoresis.

Figure S2 TGF-β protein band

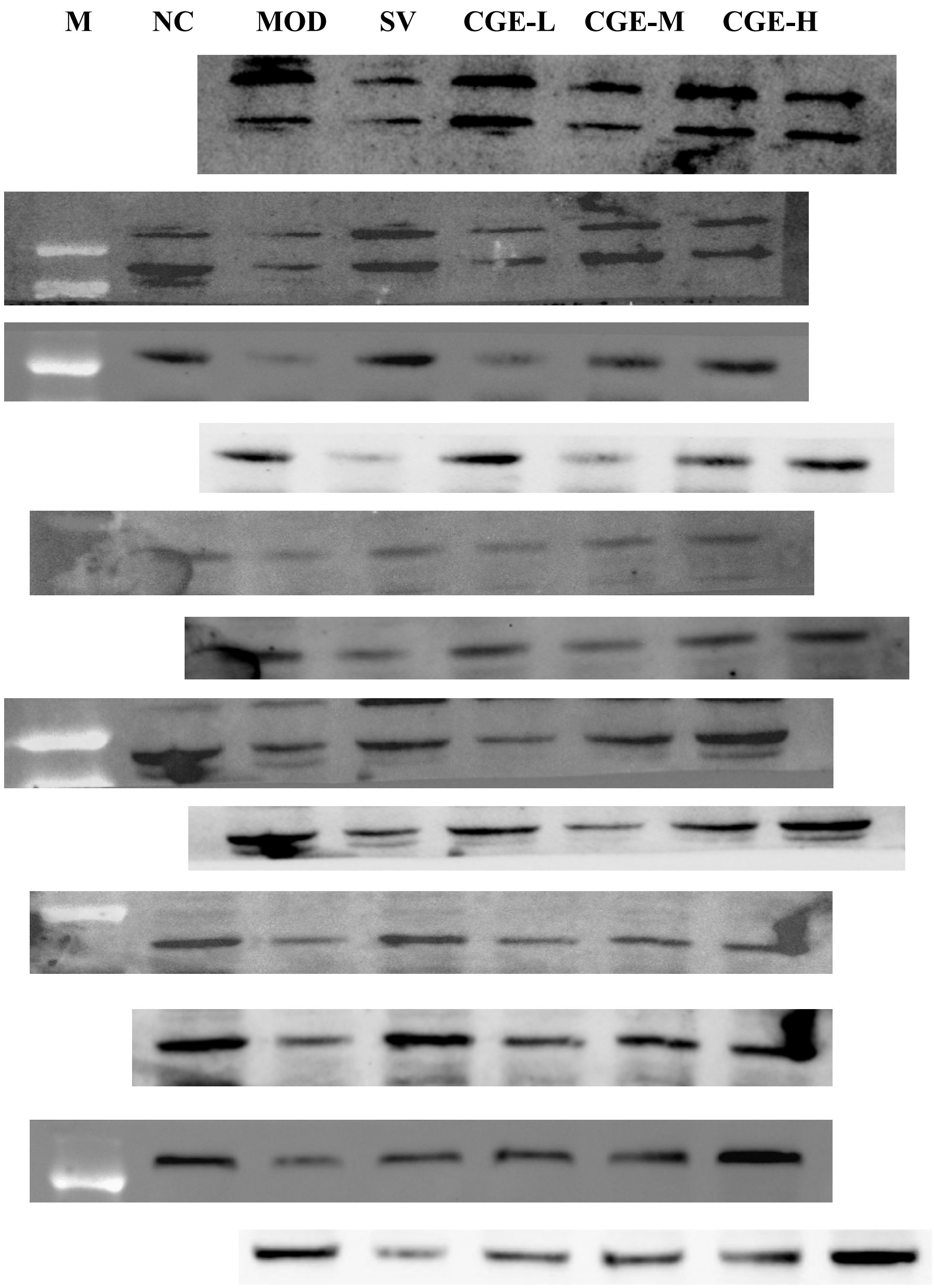

M: Marker; NC: Normal control group; MOD: Model control group; SV: Simvastatin group; CGE-L: CGE low dose group; CGE-M: CGE medium dose group; CGE: CGE high dose group.

Figure S3 GAPDH protein band

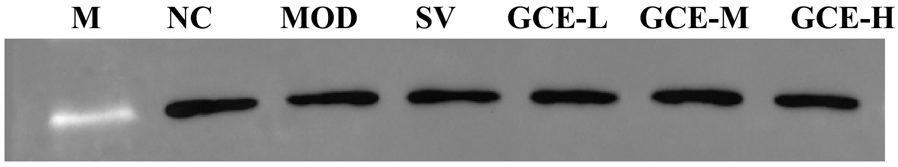

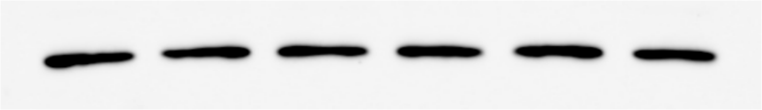

Table S17 The gray value of TGF-β protein band for each group

Number	Relative protein expression (△Gray value)					
	NC	MOD	SV	CGE-L	CGE-M	CGE-H
1	0.40	0.26	0.59	0.23	0.34	0.38
2	0.90	0.41	0.68	0.26	0.54	0.84
3	0.71	0.15	0.76	0.28	0.64	0.77
4	0.45	0.17	0.52	0.21	0.33	0.63
5	0.21	0.17	0.28	0.19	0.21	0.21
6	0.72	0.28	0.50	0.55	0.48	0.69
$\bar{x}$ ± S	0.61±0.14**	0.24±0.13	0.61±0.12**	0.32±0.14	0.53±0.17*	0.65±0.11**

Data were expressed as mean ± SE, * Significant difference from MOD group at $p<0.05$, ** and **Very significant difference from MOD group at $p<0.01$.

Figure S4 PI3K protein band

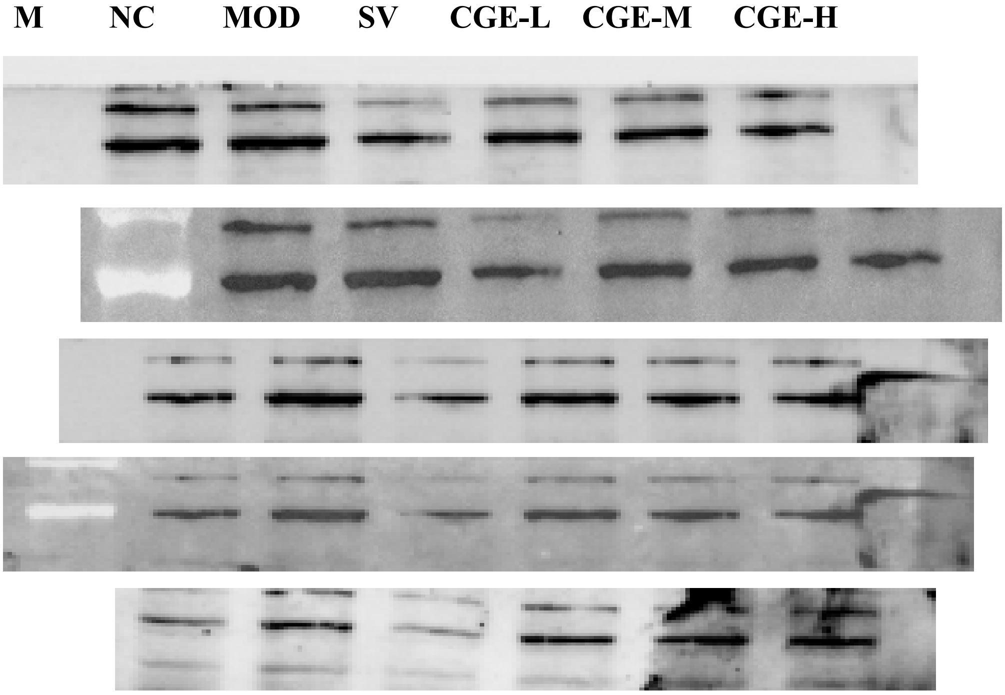

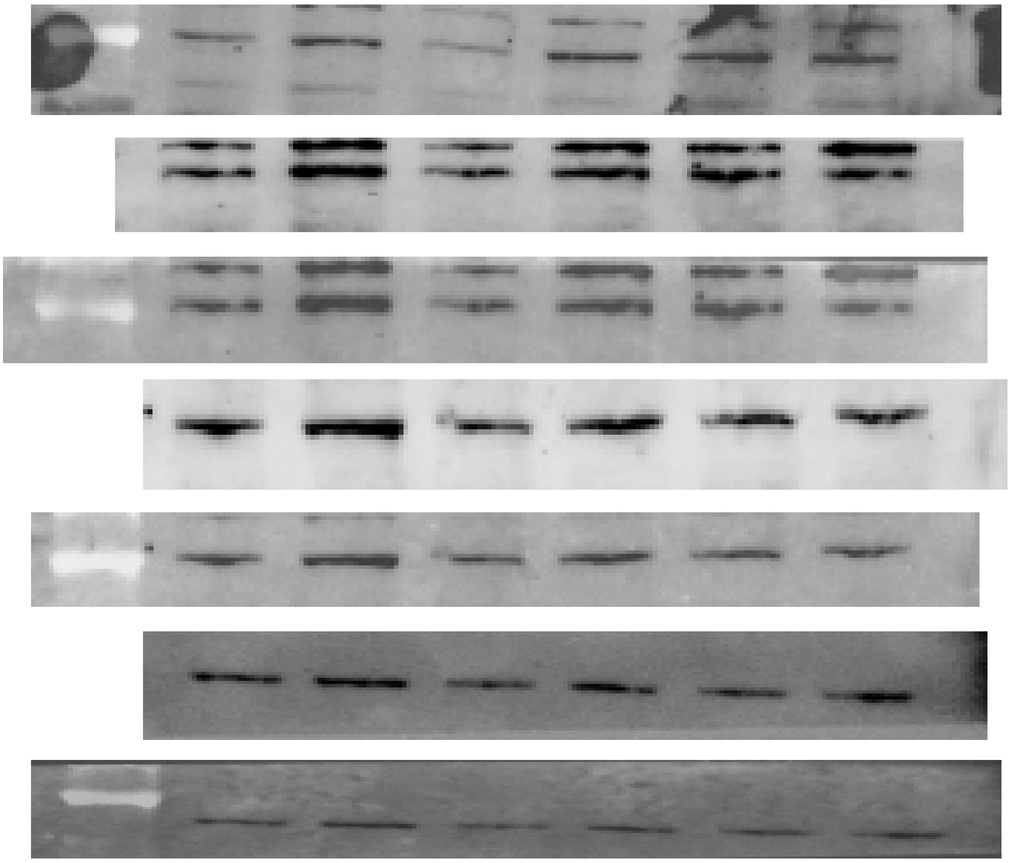

M: Marker; NC: Normal control group; MOD: Model control group; SV: Simvastatin group; CGE-L: CGE low dose group; CGE-M: CGE medium dose group; CGE: CGE high dose group.

Figure S5 GAPDH protein band

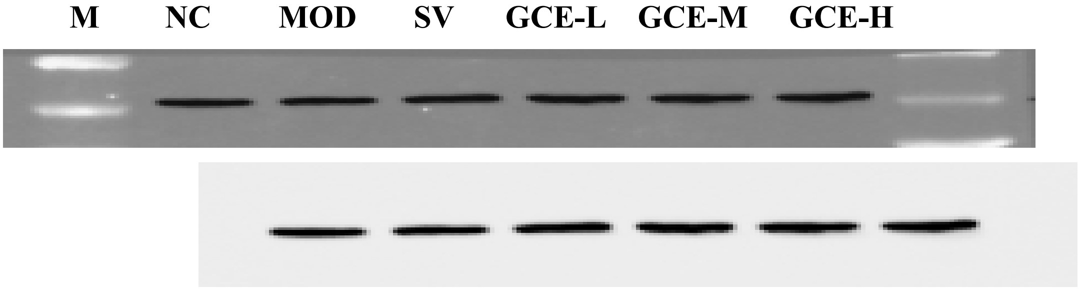

Table S18 The gray value of PI3K protein band for each group

Number	Relative protein expression (△Gray value)					
	NC	MOD	SV	CGE-L	CGE-M	CGE-H
1	0.46	1.20	0.68	0.84	0.73	0.71
2	0.59	1.00	0.42	0.75	0.63	0.62
3	0.35	0.64	0.22	0.61	0.46	0.38
4	0.60	1.05	0.43	0.72	0.65	0.51

5	0.60	0.91	0.42	0.60	0.45	0.43
6	0.34	0.42	0.19	0.29	0.22	0.27
$\bar{x}$ ± S	0.49±0.12**	0.87±0.28	0.39±0.17**	0.63±0.19*	0.52±0.18**	0.48±0.16**

Data were expressed as mean ± SE, * Significant difference from MOD group at p<0.05, ** and **Very significant difference from MOD group at p<0.01.

Figure S6 AKT 1 protein band

M NC MOD SV CGE-L CGE-M CGE-H

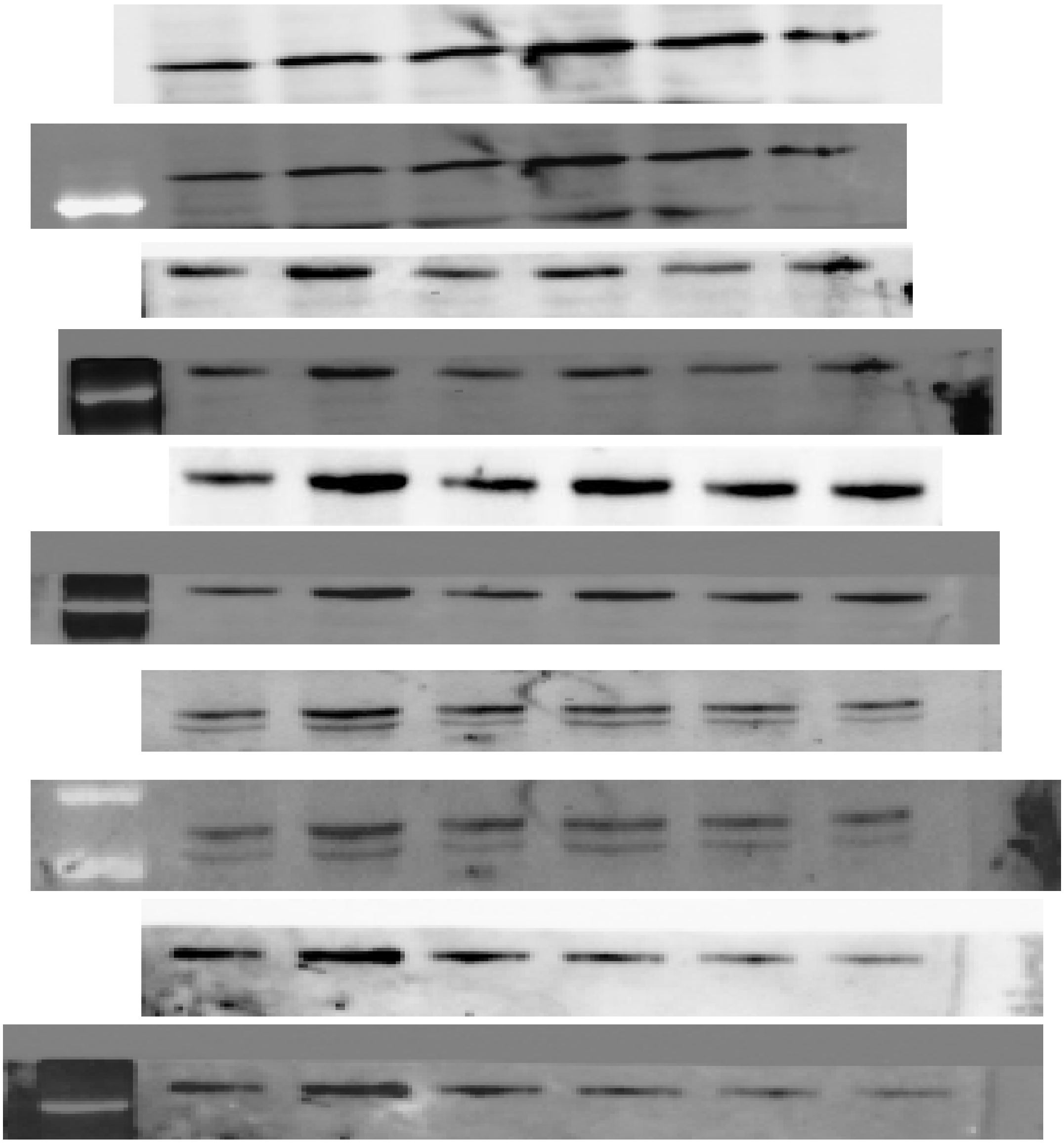

Figure S7 GAPDH protein band

M NC MOD SV GCE-L GCE-M GCE-H

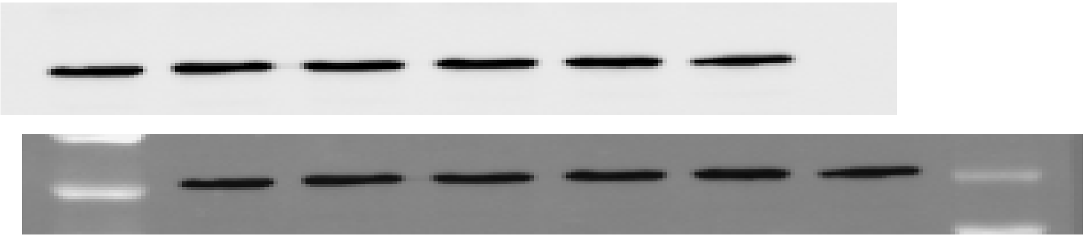

Table S19 The gray value of AKT1 protein band for each group

Number	Relative protein expression (△Gray value)					
	NC	MOD	SV	CGE-L	CGE-M	CGE-H
1	0.77	0.85	0.62	1.28	0.99	0.58
2	0.70	0.95	0.53	0.73	0.44	0.52
3	0.66	1.12	0.75	0.98	0.80	0.77
4	0.41	0.69	0.43	0.52	0.42	0.34
5	0.48	1.03	0.61	0.51	0.37	0.29
$\bar{x}$ ± S	0.60±0.15*	0.93±0.16	0.58±0.11**	0.80±0.32	0.60±0.27*	0.50±0.19**

Data were expressed as mean ± SE, * Significant difference from MOD group at $p<0.05$, ** and **Very significant difference from MOD group at $p<0.01$.

Table S20 The OD values of aorta tissues

Number	OD (λ=570nm)					
	NC	MOD	SV	CGE-L	CGE-M	CGE-H
1	0.532	0.543	0.62	0.524	0.672	0.607
2	0.42	0.477	0.433	0.588	0.533	0.599
3	0.425	0.51	0.48	0.58	0.541	0.538
4	0.633	0.655	0.592	0.546	0.632	0.58
5	0.634	0.63	0.505	0.537	0.54	0.635

Table S21 The protein assay of aorta tissues

	Protein content

Number	NC	MOD	SV	CGE-L	CGE-M	CGE-H
1	12.84217	13.205403	15.74802	12.578004	17.465112	15.318747
2	9.14382	11.026017	9.573093	14.691348	12.875193	15.054579
3	9.308925	12.11571	11.12508	14.42718	13.139361	13.040298
4	16.17729	16.903755	14.823432	13.304466	16.144272	14.42718
5	16.21031	16.07823	11.950605	13.007277	13.10634	16.243335

Dilute the original solution to a concentration of 2 μ g/μ l, 20 μ l of loading volume per well.

Figure S8 PPAR-γ and β-actin protein band

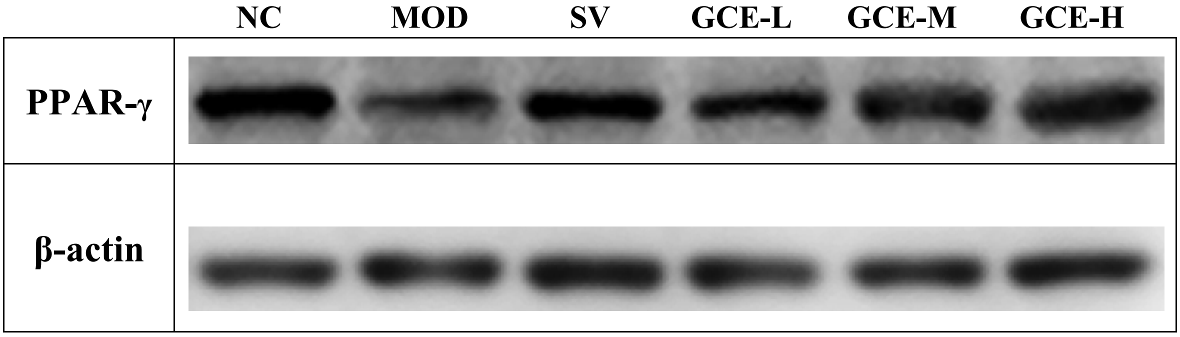

Table S22 The gray value of PPAR-γ, β-actin protein band for each group

**PPAR-γ**		Control gray values	Band gray values	Δgray values
**NC**	1	44043	78174	34131
	2	33111	60270	27159
	3	43437	72946	29509
**MOD**	1	37919	67268	29349
	2	34374	50416	16042
	3	38085	63349	25264
**SV**	1	46303	75288	28985
	2	33149	57931	24782
	3	41298	71024	29726
**GCE-L**	1	42783	62236	19453
	2	30431	52342	21911

	3	38212	64119	25907
**GCE-M**	1	43804	71785	27981
	2	30329	54688	24359
	3	40728	68311	27583
**GCE-H**	1	38163	72578	34415
	2	25429	54901	29472
	3	32713	68530	35817
β-actin		Control gray values	Band gray values	Δgray values
**NC**	1	1021846	3542438	2520592
	2	895587	3329594	2434007
	3	1269951	3989179	2719228
**MOD**	1	1277967	5840557	4562590
	2	1367050	5562279	4195229
	3	1501831	5337947	3836116
**SV**	1	1029938	3952098	2922160
	2	970583	3963682	2993099
	3	1264098	3888016	2623918
**GCE-L**	1	965725	3189227	2223502
	2	982231	2952766	1970535
	3	1167186	3552785	2385599
**GCE-M**	1	853911	3011715	2157804
	2	964737	2901637	1936900
	3	1159738	3483242	2323504
**GCE-H**	1	846873	2691754	1844881
	2	767245	2246522	1479277
	3	1007551	2938377	1930826

Table S23 The gray value of PPAR-γ protein band($\overline{x} \pm SE$)

Number	Relative protein expression (△Gray value)					
	NC	MOD	SV	CGE-L	CGE-M	CGE-H
1	1.14	0.54	0.84	0.74	1.09	1.57
2	0.94	0.32	0.70	0.94	1.06	1.68
3	0.92	0.56	0.96	0.92	1.00	1.57
$\bar{x}$ ± S	1.0±0.12***	0.47±0.13	0.83±0.13**	0.86±0.11**	1.05±0.05***	1.61±0.06***

Data were expressed as mean ± SE, * Significant difference from MOD group at p<0.01**, and ***Very significant difference from MOD group at p<0.001.

Figure S9 The expression of LXR-α protein for each group

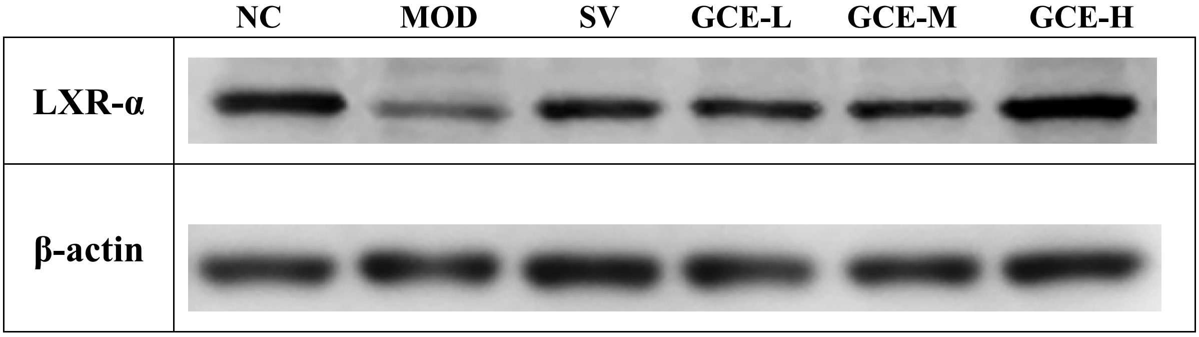

Table S24 The gray value of LXR-α, β-actin protein band for each group

LXR-α		Control gray values	Band gray values	Δgray values
NC	1	15155	41936	26781
	2	21734	53191	31457
	3	26407	57321	30914
MOD	1	17433	29481	12048
	2	23689	38772	15083
	3	27747	42873	15126
SV	1	15216	38508	23292
	2	22176	48142	25966
	3	25203	51795	26592
	1	16049	36917	20868

**GCE-L**	2	23117	46730	23613
	3	25125	50259	25134
**GCE-M**	1	16026	35927	19901
	2	23183	45292	22109
	3	26283	49115	22832
**GCE-H**	1	21565	47785	26220
	2	29277	61318	32041
	3	34626	66258	31632
β-actin		Control gray values	Band gray values	Δgray values
**NC**	1	1893569	3802012	1908443
	2	1315421	3618954	2303533
	3	870387	3029751	2159364
**MOD**	1	1301717	4146403	2844686
	2	1420465	3881253	2460788
	3	1000401	3827504	2827103
**SV**	1	1167908	3246869	2078961
	2	1111789	3332255	2220466
	3	857172	2844913	1987741
**GCE-L**	1	1130330	3425420	2295090
	2	1047497	3173002	2125505
	3	756583	2774246	2017663
**GCE-M**	1	1030528	3346789	2316261
	2	933319	3120426	2187107
	3	786562	2730705	1944143
**GCE-H**	1	861660	2078640	1216980
	2	899441	2717086	1817645
	3	687976	2354616	1666640

Table S25 The gray value of LXR-α protein band for each group

Number	Relative protein expression (△Gray value)					
	NC	MOD	SV	CGE-L	CGE-M	CGE-H
1	1.00	0.30	0.80	0.65	0.61	1.54
2	0.98	0.44	0.84	0.79	0.72	1.26
3	1.02	0.38	0.96	0.89	0.84	1.36
$\bar{x}$ ± S	1.00±0.02***	0.37±0.07	0.86±0.08***	0.78±0.12**	0.72±0.11**	1.38±0.14***

Data were expressed as mean ± SE, * Significant difference from MOD group at p<0.01**, and ***Very significant difference from MOD group at p<0.001.

Figure S10 The expression band of ABCA1 protein for each group

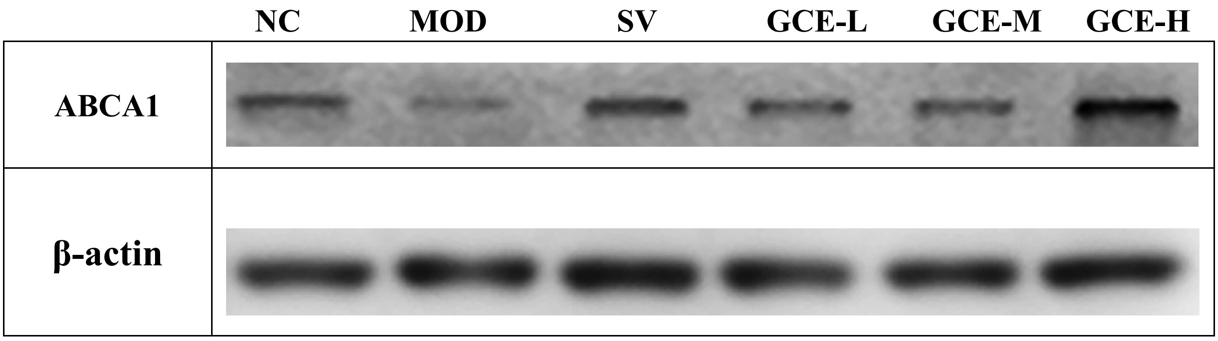

Table S26 The gray value of ABCA1, β-actin protein band for each group

ABCA1		Control gray values	Band gray values	Δgray values
**NC**	1	45486	56492	11006
	2	28644	39441	10797
	3	37306	47895	10589
**MOD**	1	49546	53769	4223
	2	31389	37126	5737
	3	40430	45268	4838
**SV**	1	45078	55470	10392
	2	28572	38768	10196
	3	36855	47552	10697

**GCE-L**	1	44170	51647	7477
	2	27871	35137	7266
	3	36537	43611	7074
**GCE-M**	1	47011	55412	8401
	2	29508	40131	10623
	3	38848	45955	7107
**GCE-H**	1	56407	65864	9457
	2	32907	47432	14525
	3	45507	58340	12833
β-actin		Control gray values	Band gray values	Δgray values
**NC**	1	1893569	3802012	1908443
	2	1315421	3618954	2303533
	3	870387	3029751	2159364
**MOD**	1	1301717	3746403	2444686
	2	1420465	3881253	2460788
	3	1000401	3827504	2827103
**SV**	1	1167908	3246869	2078961
	2	1111789	3332255	2220466
	3	857172	2844913	1987741
**GCE-L**	1	1130330	3425420	2295090
	2	1047497	3173002	2125505
	3	756583	2774246	2017663
**GCE-M**	1	1030528	3346789	2316261
	2	933319	3120426	2187107
	3	786562	2730705	1944143
**GCE-H**	1	861660	2078640	1216980
	2	899441	2717086	1817645
	3	687976	2354616	1666640

Table S27 The gray value of ABCA1 protein band for each group

Number	Relative protein expression (△Gray value)					
	NC	MOD	SV	CGE-L	CGE-M	CGE-H
1	1.13	0.34	0.98	0.64	0.71	1.52
2	0.92	0.46	0.90	0.67	0.95	1.56
3	0.96	0.33	1.05	0.68	0.71	1.50
$\bar{x}$ ± S	1.00±0.11***	0.38±0.07	0.97±0.08***	0.66±0.02**	0.79±0.14***	1.53±0.023***

Data were expressed as mean ± SE, * Significant difference from MOD group at p<0.01**, and ***Very significant difference from MOD group at p<0.001.

Figure S11. Real time-PCR amplification procedure

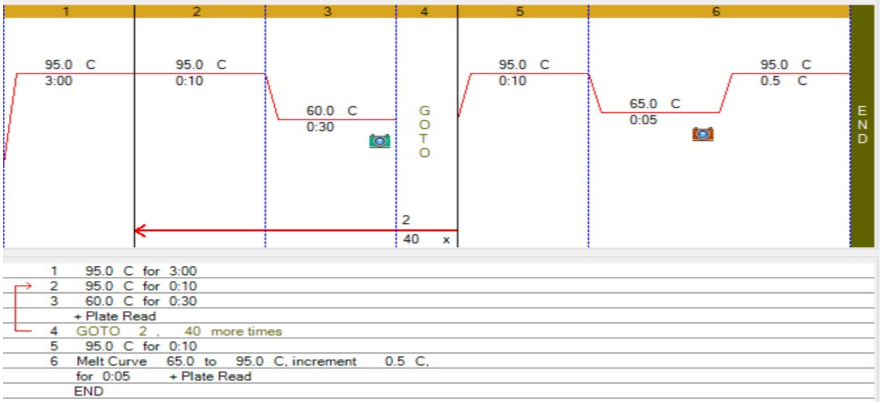

Table S28 The mRNA expression level of TGF-β、PI3K and AKT in livers (n=6)

Groups	mRNA relative expression level		
	TGF-β	PI3K	AKT
NC	1.06 ± 0.15**	1.01 ± 0.09**	1.01±0.05**
MOD	0.36 ± 0.05	1.90 ± 0.23	1.89±0.39
SV	0.96 ± 0.18**	1.26 ± 0.17**	1.15±0.23**
GCE-L	0.36 ± 0.02	1.64 ± 0.03*	1.58±0.30
GCE-M	0.85 ± 0.03**	1.35 ± 0.38**	1.30±0.37*

GCE-H	0.94 ± 0.12**	1.27 ± 0.01**	0.99±0.02**

Table S29 The mRNA expression level of PPAR γ、LXR α and ABCA1 in livers (n=6)

Groups	mRNA relative expression level		
	PPAR γ	LXR α	ABCA1
NC	1.0 ± 0.086***	1.00 ± 0.14***	1.00 ± 0.11**
MOD	0.23 ±0.04	0.46 ± 0.03	0.55 ± 0.01
SV	1.57 ± 0.14***	0.96 ± 0.16***	1.70 ± 0.08***
GCE-L	0.65 ± 0.09	1.06 ± 0.14	1.05 ± 0.13
GCE-M	1.47 ± 0.04***	1.11 ± 0.16**	1.39 ± 0.03*
GCE-H	1.53 ± 0.06***	1.56 ± 0.26***	2.19 ± 0.25***

Data were expressed as mean ± SE, * Significant difference from MOD group at $p<0.01$**, and ***Very significant difference from MOD group at $p<0.001$.

Table S30 The loading quantity of protein sample

BCA solution (μl)	0	1	2	4	8	12	16	20
X: BCA protein	0	0.05	0.1	0.2	0.4	0.6	0.8	1
Y:OD570nm	0.137	0.188	0.238	0.333	0.505	0.69	0.856	0.99

Figure S12 The protein concentration standard curve

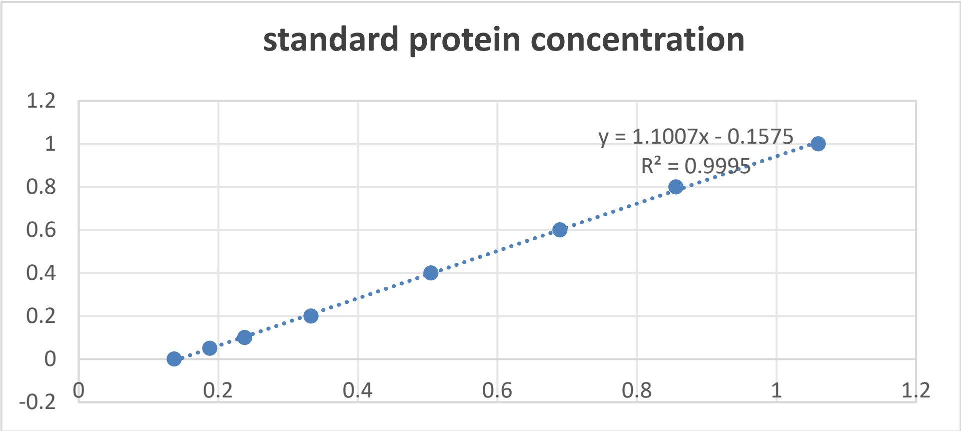

Table S31 The OD values of liver tissues

	OD (λ=570nm)

Number	NC	MOD	SV	CGE-L	CGE-M	CGE-H
1	0.651	0.632	0.853	0.854	0.854	0.617
2	0.742	0.65	0.751	0.607	0.911	0.745
3	0.78	0.712	0.733	0.685	0.746	0.746
4	0.91	0.655	0.79	0.624	0.621	0.765
5	0.622	0.64	0.621	0.627	0.595	0.822

Table S32 The protein assay of liver tissues

Number	Protein content					
	NC	MOD	SV	CGE-L	CGE-M	CGE-H
1	16.771671	16.144272	23.441913	23.474934	23.474934	15.648957
2	19.776582	16.73865	20.073771	15.318747	25.357131	19.875645
3	21.03138	18.785952	19.479393	17.894385	19.908666	19.908666
4	25.32411	16.903755	21.36159	15.880104	15.781041	20.536065
5	15.814062	16.40844	15.781041	15.979167	14.922495	22.418262

Figure S13 TGF-β protein band of mouse livers

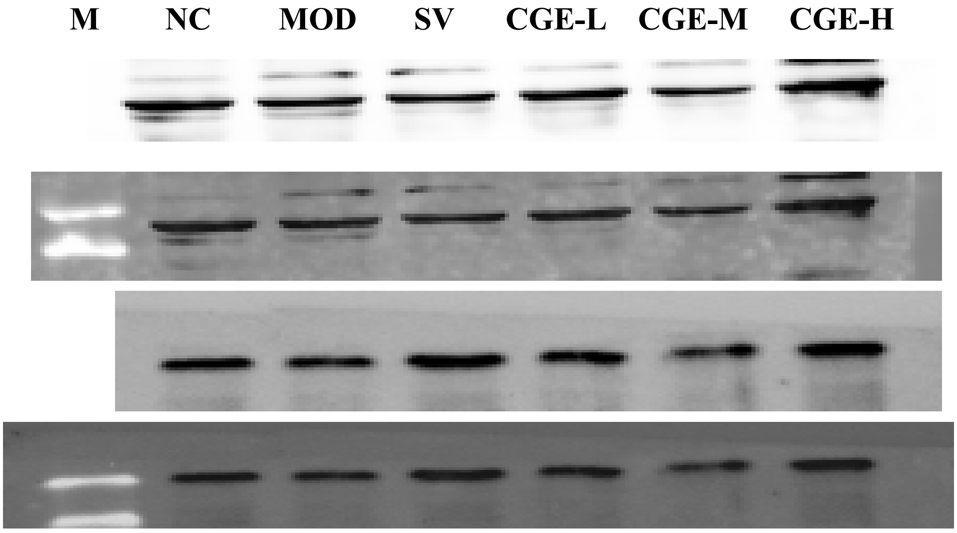

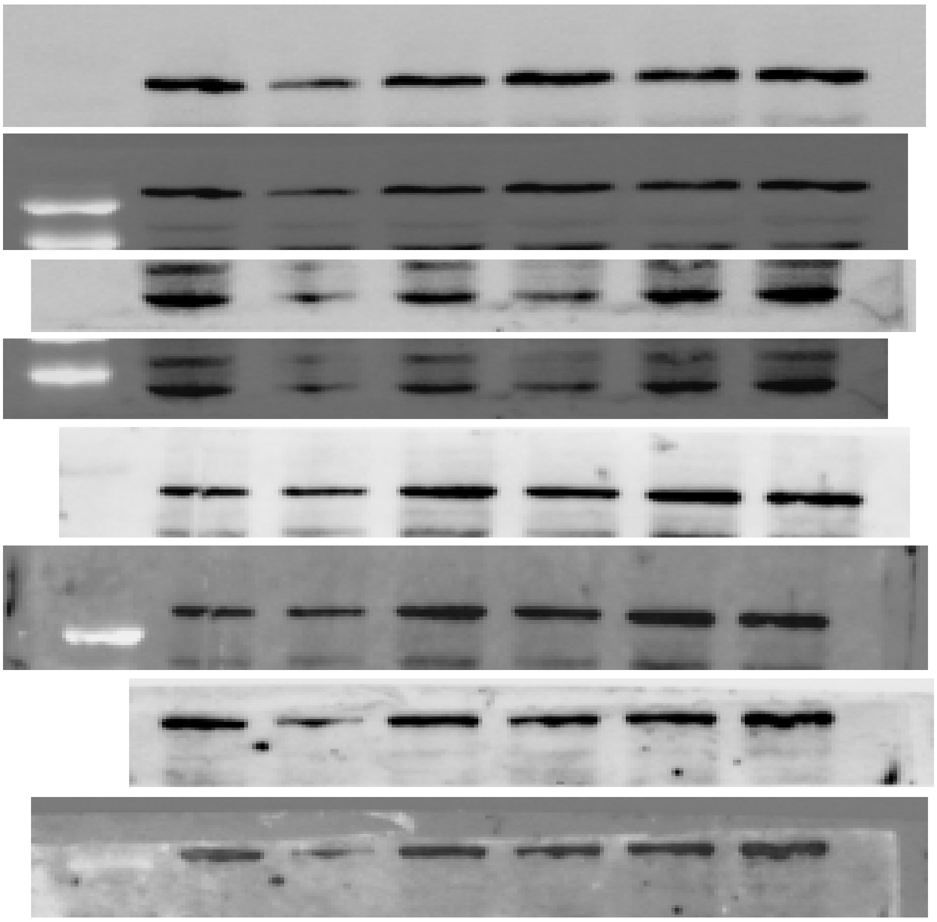

Figure S14 GAPDH protein band of mouse livers

M NC MOD SV GCE-L GCE-M GCE-H

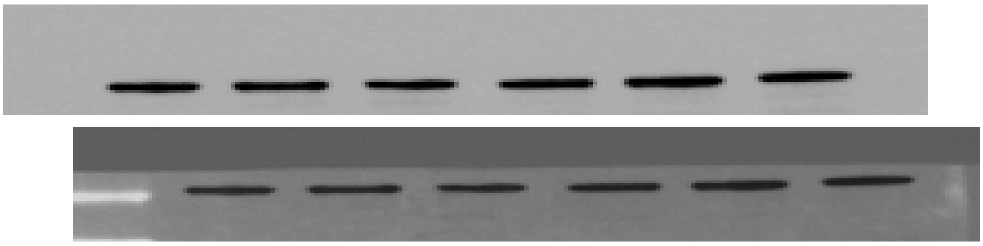

Table S33 The gray value of TGF-β protein band for each group

Number	Relative protein expression (△Gray value)					
	NC	MOD	SV	CGE-L	CGE-M	CGE-H
1	1.59	1.10	1.38	1.30	1.00	1.07
2	1.07	0.87	1.36	0.90	0.94	1.29
3	1.42	0.62	1.30	1.39	1.22	1.53
4	1.68	0.60	1.36	0.84	1.43	1.68

5	1.15	0.94	1.80	1.25	1.47	1.74
6	1.48	0.69	1.71	1.40	1.61	1.75
$\bar{x}$ ± S	1.40±0.24**	0.80±0.19	1.48±0.21**	1.18±0.24*	1.27±0.27**	1.51±0.27**

Data were expressed as mean ± SE, * Significant difference from MOD group at p<0.01**, and ***Very significant difference from MOD group at p<0.001.

Figure S15 PI3K protein band

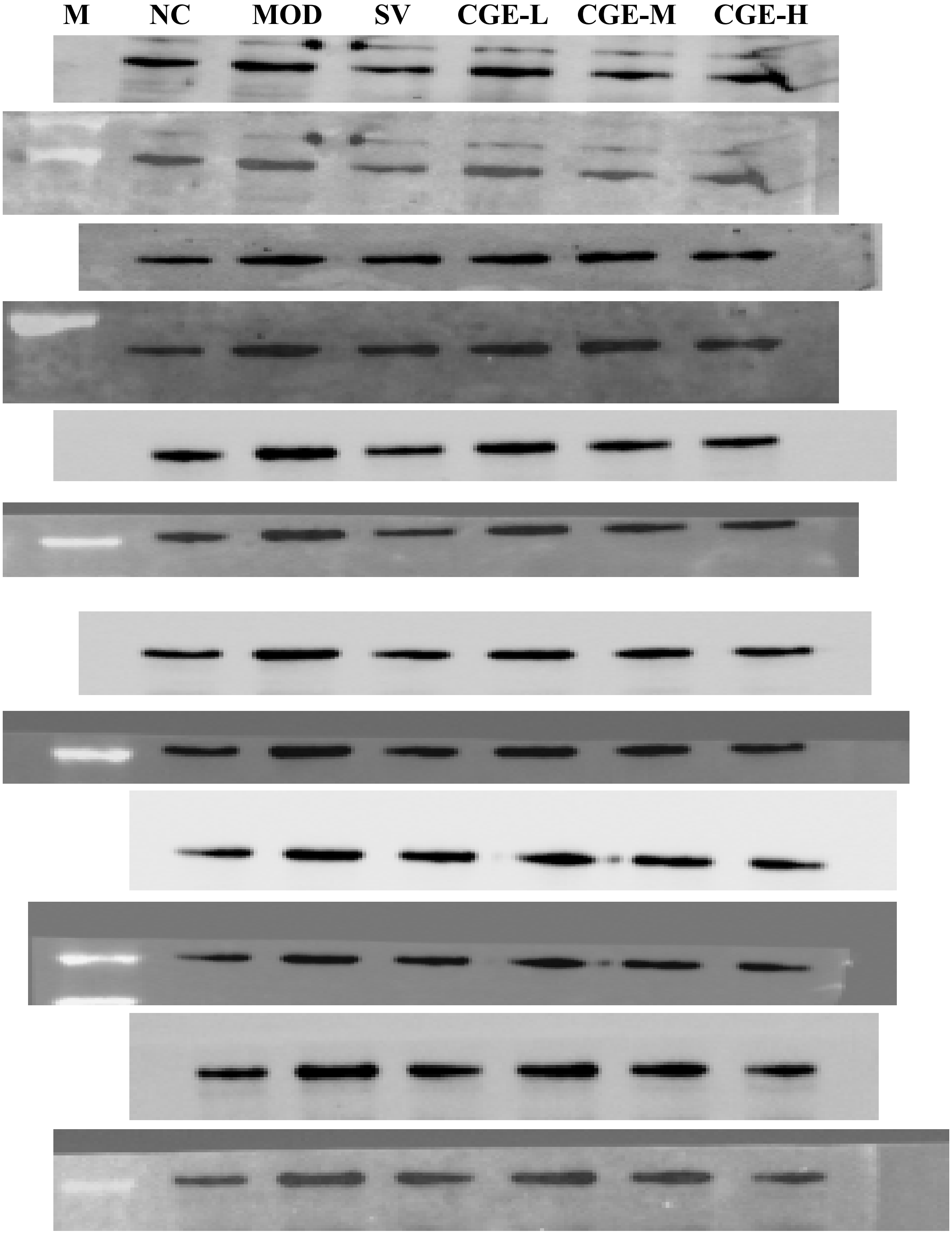

Figure S16 GAPDH protein band

M NC MOD SV GCE-L GCE-M GCE-H

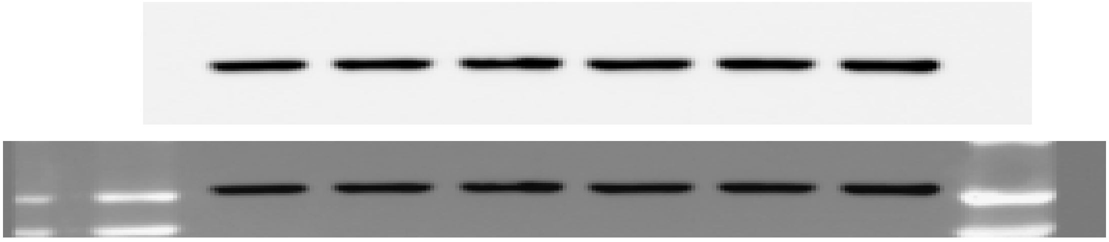

Table S34 The gray value of PI3K protein band for each group

Number	Relative protein expression (△Gray value)					
	NC	MOD	SV	CGE-L	CGE-M	CGE-H
1	0.62	0.80	0.55	0.80	0.61	0.56
2	0.43	0.72	0.56	0.60	0.62	0.50
3	0.77	1.14	0.73	1.01	0.83	0.72
4	0.70	1.02	0.69	0.93	0.79	0.66
5	0.76	1.20	0.99	1.11	1.07	0.79
6	0.62	0.96	0.70	0.90	0.79	0.54
$\bar{x}$ ± S	0.65±0.12**	0.97±0.18	0.70±0.15**	0.89±0.17	0.78±0.16*	0.62±0.11**

Data were expressed as mean ± SE, * Significant difference from MOD group at p<0.01**, and ***Very significant difference from MOD group at p<0.001.

Figure S17 AKT1 protein band

M NC MOD SV CGE-L CGE-M CGE-H

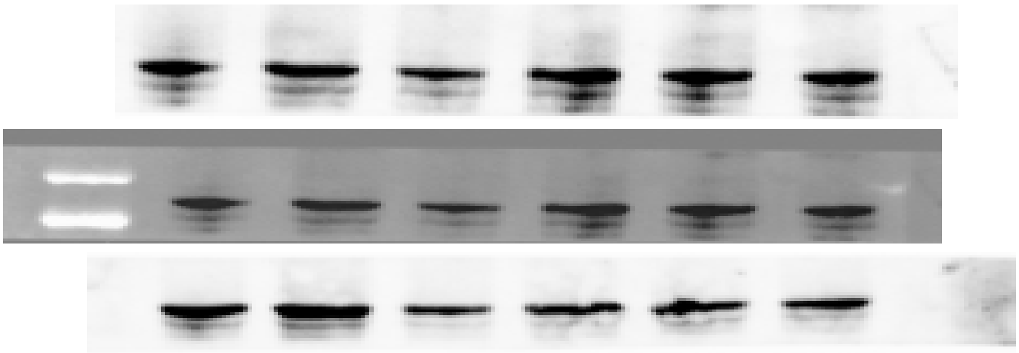

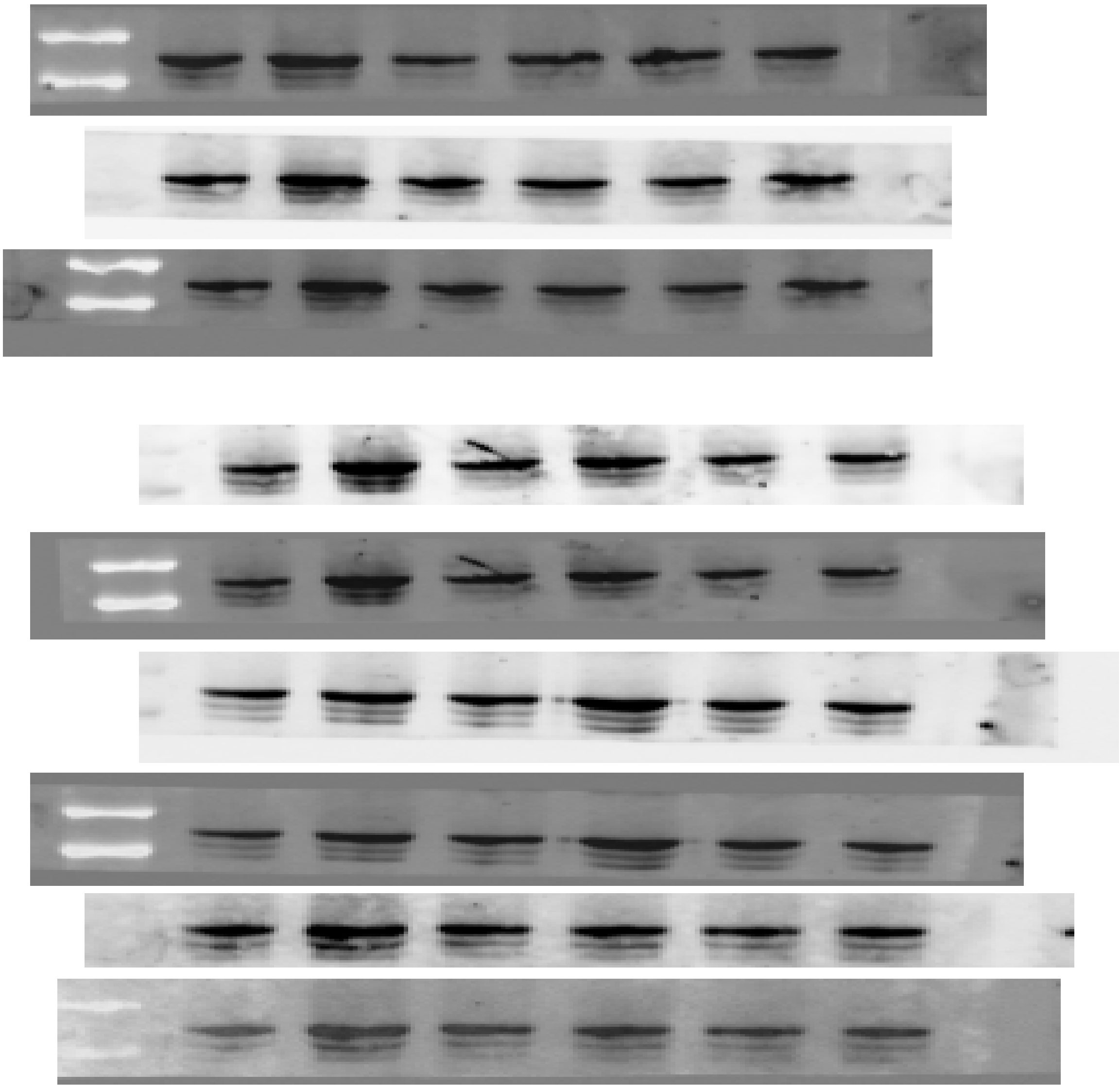

Figure S18 GAPDH protein band

M NC MOD SV GCE-L GCE-M GCE-H

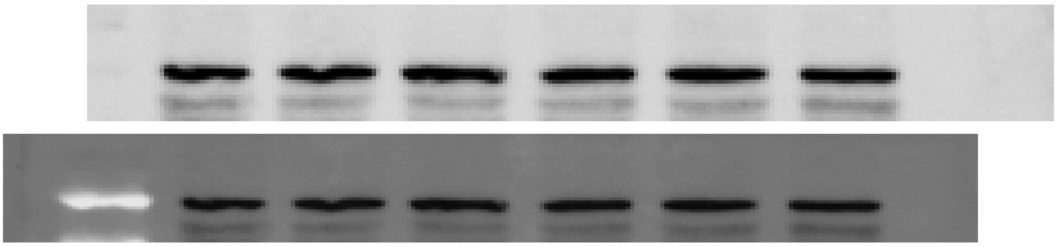

Table S35 The gray value of AKT1 protein band for each group10

Number	Relative protein expression (△Gray value)					
	NC	MOD	SV	CGE-L	CGE-M	CGE-H
1	0.69	1.18	0.84	1.22	1.11	1.07

2	0.80	1.00	0.72	1.06	0.96	0.76
3	0.81	1.22	0.58	1.02	0.78	0.66
4	0.72	1.17	0.71	1.13	0.89	0.78
5	0.62	1.06	0.73	0.90	0.78	0.61
6	0.53	1.26	0.63	0.85	0.73	0.65
$\bar{x} \pm S$	0.69±0.10**	1.14±0.09	0.70±0.08**	1.03±0.13	0.87±0.14**	0.75±0.16**

Data were expressed as mean ± SE, * Significant difference from MOD group at p<0.05, ** and **Very significant difference from MOD group at p<0.01.

Figure S19 PPAR-γ protein band

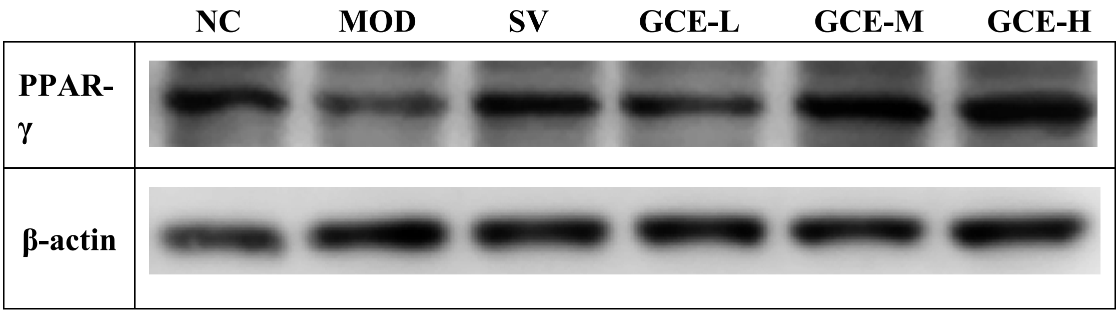

Table S36 The gray value of PPAR-γ, β-actin protein band for each group

**PPAR-γ**		Control gray values	Band gray values	Δgray values
**NC**	1	155118	237024	81906
	2	156602	243083	86481
	3	164057	254882	90825
**MOD**	1	137651	192254	54603
	2	139066	187246	48180
	3	134785	173237	38452
**SV**	1	152335	230682	78347
	2	144040	226058	82018
	3	144520	200378	55858
**GCE-L**	1	136872	188410	51538
	2	131729	196692	64963

	3	141166	179987	38821
**GCE-M**	1	174427	254135	79708
	2	174771	249899	75128
	3	169941	237013	67072
**GCE-H**	1	161132	251134	90002
	2	150249	247619	97370
	3	160444	245598	85154
β-actin		Control gray values	Band gray values	Δgray values
**NC**	1	962083	3237936	2275853
	2	926105	3444517	2518412
	3	710078	2990987	2280909
**MOD**	1	1070630	4552465	3481835
	2	1078617	4930832	3852215
	3	939403	4191766	3252363
**SV**	1	849263	3085221	2235958
	2	957863	3585739	2627876
	3	760335	3002117	2241782
**GCE-L**	1	826912	2805440	1978528
	2	837422	3482546	2645124
	3	725195	2740821	2015626
**GCE-M**	1	744382	2596418	1852036
	2	863802	2805891	1942089
	3	801569	2681858	1880289
**GCE-H**	1	717165	2335815	1618650
	2	711993	2515761	1803768
	3	560800	2227024	1666224

Table S37 The gray value of PPAR-γ protein band for each group

	Relative protein expression (△Gray value)

Number	NC	MOD	SV	CGE-L	CGE-M	CGE-H
1	0.99	0.19	1.62	0.75	1.45	1.58
2	0.92	0.21	1.42	0.59	1.52	1.47
3	1.09	0.27	1.69	0.62	1.45	1.54
$\bar{x}$ ± S	1.00±0.09***	0.23±0.04	1.57±0.14***	0.65±0.09***	1.47±0.04***	1.53±0.06***

Data were expressed as mean ± SE, * Significant difference from MOD group at p<0.01, * *and ***Very significant difference from MOD group at p<0.001.

Figure S20 The expression band of LXR-α protein for each group

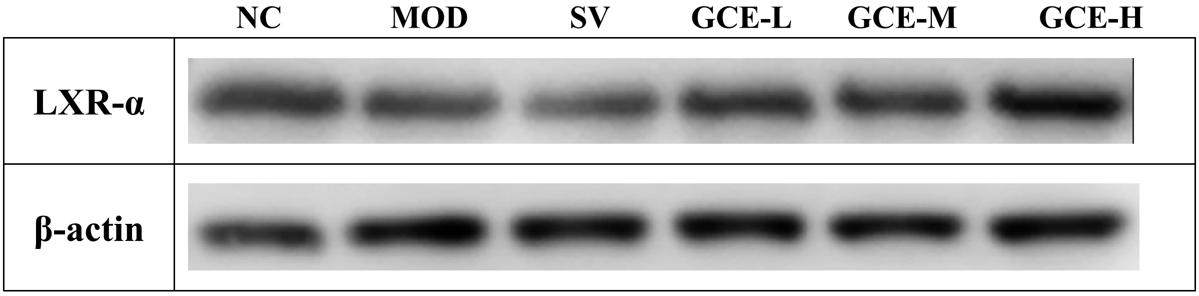

Table S38 The gray value of LXR-α, β-actin protein band for each group

**LXR-α**		Control gray values	Band gray values	Δgray values
**NC**	1	26390	69113	42723
	2	21053	58273	37220
	3	18787	58083	39296
**MOD**	1	24368	60068	35700
	2	19376	50934	31558
	3	19804	50501	30697
**SV**	1	21151	54382	33231
	2	20903	56626	35723
	3	18150	56893	38743
**GCE-L**	1	25033	67810	42777
	2	21505	56932	35427

	3	20518	57674	37156
**GCE-M**	1	30787	66834	36047
	2	27479	56022	28543
	3	23420	56787	33367
**GCE-H**	1	27891	78314	50423
	2	26590	76362	49772
	3	23101	67052	43951
β-actin		Control gray values	Band gray values	Δgray values
**NC**	1	962083	3237936	2275853
	2	926105	3544517	2518412
	3	710078	2990987	2280909
**MOD**	1	1070630	5452465	3481835
	2	1078617	5230832	3852215
	3	939403	5191766	3252363
**SV**	1	849263	2995221	2235958
	2	957863	3585739	2627876
	3	760335	2802117	2241782
**GCE-L**	1	826912	2805440	1978528
	2	837422	3482546	2645124
	3	725195	2740821	2015626
**GCE-M**	1	744382	2496418	1852036
	2	863802	2705891	1942089
	3	801569	2481858	1880289
**GCE-H**	1	717165	2335815	1618650
	2	711993	2915761	1803768
	3	560800	2327024	1666224

Table S39 The gray value of LXR-α protein band for each group

Number	Relative protein expression (△Gray value)					
	NC	MOD	SV	CGE-L	CGE-M	CGE-H
1	1.12	0.49	0.93	1.29	1.23	1.86
2	0.85	0.47	0.81	0.80	0.93	1.35
3	1.03	0.43	1.13	1.10	1.19	1.49
$\bar{x}$ ± S	1.00±0.14	0.46±0.03	0.96±0.16	1.06±0.14	1.11±0.16	1.56±0.26

Data were expressed as mean ± SE, * Significant difference from MOD group at p<0.01, **and ***Very significant difference from MOD group at p<0.001.

Figure S21 The expression band of ABCA1 protein for each group

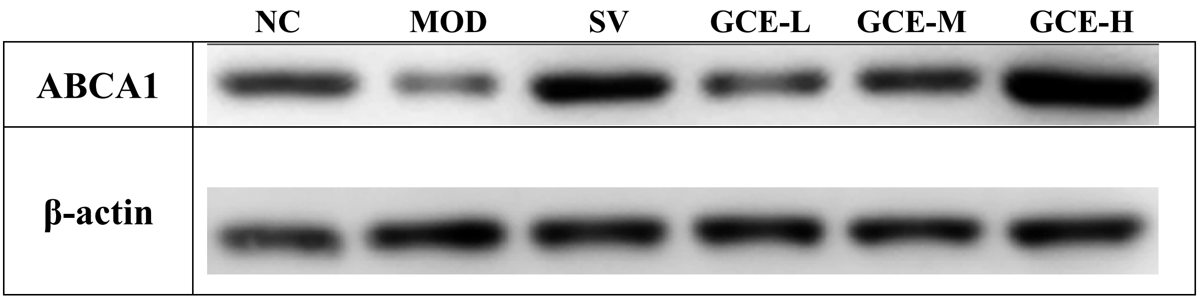

Table S40 The gray value of ABCA1, β-actin protein band for each group

ABCA1		Control gray values	Band gray values	Δgray values
NC	1	9633	50951	41318
	2	9128	48374	39246
	3	9539	47610	38071
MOD	1	8307	25938	17631
	2	8317	26988	18671
	3	8530	25068	16538
SV	1	14103	72197	58094
	2	8169	67805	59636
	3	9606	57805	48199
	1	9789	46898	37109

GCE-L	2	13491	43380	29889
	3	13453	43652	30199
GCE-M	1	13805	57416	43611
	2	5601	53110	47509
	3	7104	49307	42203
GCE-H	1	29722	88974	59252
	2	24950	81550	56600
	3	25700	80504	54804
β-actin		Control gray values	Band gray values	Δgray values
NC	1	962083	3237936	2275853
	2	926105	3544517	2518412
	3	710078	2990987	2280909
MOD	1	1070630	5452465	3481835
	2	1078617	5230832	3852215
	3	939403	5191766	3252363
SV	1	849263	2995221	2235958
	2	957863	3585739	2627876
	3	760335	2802117	2241782
GCE-L	1	826912	2805440	1978528
	2	837422	3482546	2645124
	3	725195	2740821	2015626
GCE-M	1	744382	2496418	1852036
	2	863802	2705891	1942089
	3	801569	2481858	1880289
GCE-H	1	717165	2335815	1618650
	2	711993	2915761	1803768
	3	560800	2327024	1666224

Table S41 The gray value of ABCA1 protein band for each group

Number	Relative protein expression (△Gray value)					
	NC	MOD	SV	CGE-L	CGE-M	CGE-H
1	1.07	0.55	1.72	1.17	1.39	2.43
2	0.87	0.53	1.60	0.91	1.42	1.94
3	1.06	0.56	1.77	1.07	1.36	2.20
$\bar{x}$ ± S	1.00±0.11**	0.55±0.01	1.70±0.08***	1.05±0.10**	1.34±0.03***	2.19±0.25***

Data were expressed as mean ± SE, * Significant difference from MOD group at p<0.01, **and ***Very significant difference from MOD group at p<0.001.
